# Methodological Proposal for the Adaptation of the Living with Long-Term Conditions Scale to the Family Caregiver

**DOI:** 10.3390/nursrep14010041

**Published:** 2024-02-27

**Authors:** Patricia Marín-Maicas, Mari Carmen Portillo, Silvia Corchón, Leire Ambrosio

**Affiliations:** 1 Faculty of Health Science, Valencian International University, 46002 Valencia, Spain; pmarinm@universidadviu.com; 2 Faculty of Nursing and Chiropody, University of Valencia, 46010 Valencia, Spain; 3 NIHR ARC Wessex, School of Health Sciences, University of Southampton, Southampton SO17 1BJ, UK; m.c.portillo-vega@soton.ac.uk (M.C.P.); l.ambrosio-gutierrez@soton.ac.uk (L.A.)

**Keywords:** living with long-term conditions, methodological approach, family caregiver, tool

## Abstract

(1) Background: Living with long-term conditions affects both patients and family caregivers. To obtain a more complete overview of this phenomenon, a measurement instrument is needed that includes both perspectives. The aim is to adapt a scale to family caregivers of individuals with long-term conditions. (2) Methods: A methodological proposal is presented that illustrates the adaptation of the EC-PC scale to the family caregiver. Three phases are proposed: adaptation of the items, panel of experts, and pre-test. (3) Results: In the adaptation phase, the items from the original EC-PC were modified to adapt them to the family caregiver, and new items were added associated with the differences in living with LTC from the perspective of family caregivers. In the panel of experts phase, a universal agreement was reached related to the clarity, relevance, and essentiality of the items included. In the pre-test phase, the content of the scale was verified quantitatively and qualitatively. (4) Conclusions: The content of the items of version 5 of the EC-PC-Family showed a high index of inter-judge agreement. When a phenomenon affects both patients and their environment, such as living with LTC, it is necessary to include both perspectives in the measurement tools.

## 1. Introduction

Living with long-term conditions (LTCs) can be defined as a multi-dimensional phenomenon that encompasses different dimensions such as acceptance, coping, self-management, integration, and adaptation to the disease [1]. These five dimensions are dynamic and vary depending on the many characteristics of a person [1,2]. However, living with an LTC not only affects the person with the disease but also affects the immediate family environment [3,4,5,6]. Therefore, living with LTCs is not only important for the person affected, but the family caregiver as well. The meaning of living with an LTC, and the dimensions that compose it, are mostly similar between the people with LTC and the family caregivers [7]. However, two of its dimensions, self-management and integration, have different meanings and applications [7]. The self-management of the family caregivers not only refers to the management of the LTCs but also to their own health as family caregivers. On its part, integration acquires a broader meaning, to include the management of LTCs within the family [7]. In this sense, despite the family caregiver living with the LTCs in a similar manner as the patient, it is necessary to include these differences in the measurement instruments utilized to comprehensively determine the degree of people living with the LTCs. Thus, this could optimize the management of the LTCs [6], as suggested by the WHO [8], when indicating the fundamental need to invest in better management of the LTCs at the global scale.

### Background

Caring for people living with LTCs has a negative impact on the quality of life of caregivers, affecting their psychological, emotional, social, physical, and financial well-being [9,10]. Evaluating how the family caregivers live with LTC can provide social health professionals with important information when planning interventions that favor better living [7]. Therefore, it would be useful to have a tool available that allows for objectifying the degree to which people live with LTCs, for both the individuals affected by LTCs and the family caregiver. Presently, many scales exist that allow for measuring different concepts in family caregivers. For example, some scales are available to evaluate the level of overload due to the care, the quality of life, or the repercussions of the care on health or well-being (i.e., Caregiver Quality of Life Cystic Fibrosis (CQOLCF) Scale, Family Caregiver Quality of Life (FAMQOL) Scale) [11], and other scales even exist related to some of the domains that shape the concept of living, such as the case of coping or self-care (i.e., COPE-48 or Cancer Worry Scale (CWS)) [11]. However, although an instrument exists that measures the degree of living with LTC, according to the people with LTCs the “Living with Chronic Illness Scale” (EC-PC) [1,11], no instrument was found that measures the degree of living with LTCs of family caregivers that includes all the dimensions that shape said process [12]. This gap in the literature, in relation to measurement instruments that evaluate how the family caregiver experiences living with LTCs, is the common thread in this work. Based on an instrument created for people living with LTCs (EC-PC) [11], an adaptation of this scale was planned to extend the reach of the instrument to family caregivers. Numerous recommendations exist on the process of adaptation of a scale to a context different from which it was created [13], but the available guides only refer to the methodological process for their transcultural adaptation, and especially to the guidelines established that allow the use of the scale in a language that is different from the one used for its creation. However, no specific system with the necessary methodological steps to be taken for the adaptation of the EC-PC to the family caregiver was found. Thus, the good practices described by Heggestad et al. [14] for the modification of instruments were followed in the present article. Lastly, despite this methodological gap, different scales that were originally aimed toward patients have been posteriorly adapted to family caregivers (i.e., Best Findings Scale (BF), Instrumento de Evaluación de la experiencia del Paciente Crónico (IEXPAC), Psychosocial Adjustment to Illness Scale (PAIS), Patient-Professional Interaction Questionnaire (PPIQ)), [15,16,17,18] although the detailed and methodological process of adaptation followed by the authors was not described. Therefore, the aim of this study is to present a detailed description of the methodological process of adapting the EC-PC to the family caregiver (EC-PC-Family), providing transparency to the process followed to construct a robust instrument, to contribute towards filling the gap found in the scientific literature.

## 2. Materials and Methods

**Figure 1:**
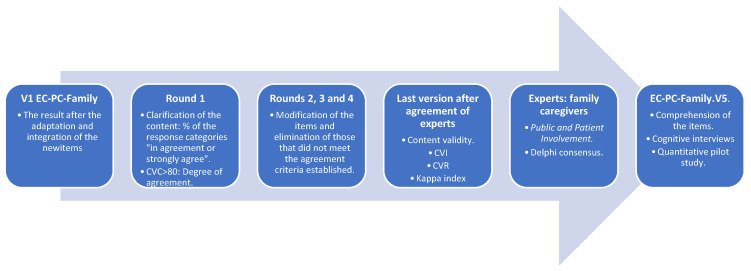
Methodological process conducted for the adaptation, from V1 to the final version (V5) of the EC-PC-Family. Created by the authors.

A methodological article is presented that illustrates the process of adapting the EC-PC scale to the family caregiver (EC-PC-Family). For the present work, the good practices described by Heggestad et al. [14] were followed for the modification of the validated instrument, which points to the need to describe and justify all the modifications included with respect to the original instrument. The study was conducted in three phases: first place, the adaptation of the EC-PC to the family caregiver to create the EC-PC-Family. In the second place, the consensus of experts was sought through a Delphi method to obtain the content validity of the items [19,20]. Lastly, a pre-test of the instrument created was conducted. The detailed process followed is shown in Figure 1.

The methodological aspects of each phase are described below:

Phase 1: Adaptation of the EC-PC to the family caregiver. To adapt the EC-PC to the family caregivers, the items that had to be modified, as they referred to the person with LTC in first person, were selected. Next, after conducting a bibliographical review to understand the meaning of living with LTC from the perspective of family caregivers [7], new items were added considering the meaning of living with LTC according to family caregivers. This adaptation work was performed by a single researcher and was presented to the group for consensus (Phase 2).

Phase 2: Panel of experts. The preliminary version of the scale was distributed to a group of experts to consult and attain consensus about the adaptation of the items [21] (Stone, 1993) through the use of the Delphi method. This method of group decision allows us to discover the opinion of a group of experts about a subject in a structured manner through a questionnaire, in which the elimination or inclusion of items was conducted through agreement [19,20,21,22,23,24,25]. This group of experts analyzed the dimensionality of the first version of the questionnaire (EC-PC-Family V1) and the adaptation of its items, individually assessing the convergence of the subject [26]. Given the heterogeneity of the existing literature with respect to the application of the Delphi method in health studies, the present work followed the specific recommendations by Diamond et al. [27], which established the three key methodological criteria that Delphi studies must contain: objective, participants, and process. These are detailed below:

1. Objective of the Delphi study: It is indispensable to clearly and specifically establish the objective of the Delphi study. In the present study, the objective was to find the essential, clear, and relevant elements that constitute the process of living with LTC from the perspective of the family caregivers. These elements were identified through an integrative review that was previously conducted [7].
2. Delphi study participants: The adequate selection of the participants in a Delphi study is a determining factor, although there is no consensus on the recommended number in the selection of experts to be included [19,23,24]. Romero-Collado [24] includes results similar to Carretero-Dios and Pérez in [24], who argued for a minimum of 3 experts; on their part, Yánez and Cuadra (2008) in [24] recommend between 10 and 18, and on the contrary, Humphrey-Murto [20] recommend a minimum of 6 and more than 12 in the case that the experts come from the same discipline, and lastly, Toronto in [24] considers that 12 to 20 experts is sufficient. Therefore, given the lack of consensus in the literature consulted, the guidelines used in the development of the original EC-PC scale [1] were followed. These were the selection of a group of experts that met the following inclusion criteria: post-graduate/doctorate degree; professional experience of more than 10 years in the area of health or social care, teaching, and/or research; and the ability to provide comprehensive opinions and suggestions, and motivation for participating in the study. In the present study, 12 healthcare professionals were included (M = 25%; F = 75%), including experts in the care of chronic diseases (50%), psychometry (25%), and family caregivers (25%). Two had professional experience and postgraduate education in social intervention psychology. Considering the area of work, 66.6% were associated with areas of teaching and research, and 33% with the area of healthcare, with 91.6% having a PhD in their specialty, and 8.3% a master’s degree. More specifically, the following were included: two psychologists who were experts in chronic diseases, a social care psychologist who was an expert in psychometry, a family doctor who was an expert in chronic diseases, four nurses who were experts in chronic diseases, a nurse who specialized in psychometry, two nurses who specialized in family caregivers, and a social care psychologist who was an expert in family caregivers.
3. Delphi study process: The Google Forms (eDelphi) tool was used for this. The questionnaire was structured into various sections. The first section contained a brief description of the study, the name of the project and its aim, the contact information of the principal researcher, in case any doubts arose during the process and the consent for their free participation. The second part included sociodemographic data of the individuals polled (age, profession, education, years of experience, area). The third part included the initial list of the items in the scale [19], with the possibility of scaled responses, considering the degree of adaptation for the inclusion of the item, which oscillated from 1 to 5, with 1 being “Not adequate”, and 5 “Very adequate”. The last section was reserved for the experts to provide suggestions, observations, or any related matter. The qualitative data provided by the experts were grouped according to themes in order to include the necessary modifications in the EC-PC-Family scale throughout the different versions created, including the perceptions, suggestions, or modifications proposed [12]. After four rounds with the experts, once an agreement was reached, the questionnaire was sent to a group of family caregiver representatives (*n* = 6), selected ad hoc to promote the active participation of the population of interest: public and patient involvement (PPI) [28]. This family caregiver group of PPI was composed of 2 men (33.3%) and 4 women (66.6%), with a mean age of 61 years old (±17.5), of which 4/6 (66.6%) had played the role of family caregiver for less than 3 years, while 33.3% had played the role for more than 3 years. In the first round, to clarify the suitability of the content, the measurements oriented towards the identification of the degree of agreement between the experts were used as criteria. More specifically, two criteria were utilized: in the first place, the percentage of one or some of the response categories. This criterion was used in the case of scalable questions, in which two contiguous categories can be considered [19]; an item was accepted if it obtained a score such as in agreement (4) or very much in agreement (5) from 70% of the experts [20]. In the second place, the content validity coefficient (CVC) > 80 was utilized, specifically designed to assess the degree of agreement with respect to each of the different items and the instrument in general [29], associating the error assigned to each item to minimize the possible bias introduced by any of the judges [30]. Next, to evaluate the evidence of the expert’s judgment in relation to the final content of the scale, the content validity was measured. Validity is defined as the extent to which any instrument measures what is intended [21]. For this, two empirical measurements were utilized: the Content Validity Index (Item-CVI and Scale-CVI), and Content Validity Reason (CVR) [20,29,30,31]. The Item-CVI (I-CVI) is computed as the number of experts providing a rating of “very relevant” for each item divided by the total number of experts. Values range from 0 to 1: when I-CVI > 0.79 the item is relevant, between 0.70 and 0.79 the item needs revisions, and if the value is below 0.70 the item is eliminated [31]. Likewise, the Scale-CVI (S-CVI) was obtained using the universal agreement method (UA), dividing the number of items that have obtained a “very relevant” rating by experts (S-CVI/UA). Values ranging from S-CVI/UA ≥ 0.8 have an excellent content validity [32]. The CVR was calculated for each item of the instrument, considering those with a CVR > 0.59 as essential items. This value, according to Lawshe [33], is determined as a function of the number of experts who participated. Although the CVI is commonly used to estimate content validity, Wynd, Schmidt, and Schaefer [34] suggest that a Kappa statistic must also be associated, aside from the CVI, to avoid agreement by chance. Kappa is calculated with the following formula: K = (I-CVI − Pc)/(1 − Pc), where Pc = [N!/A! (N − A)!] × 0.5 N. In this formula, Pc = the probability of fortuitous agreement; N = number of experts; and A = number of experts who agree that the subject is relevant. The results of the process are also shown as scores, frequencies, and/or percentages in each response category, as well as dispersion measurements and means.

Phase 3: Pre-test of the instrument. A qualitative and quantitative pilot study was conducted [35]. In the first place, a qualitative pre-test was conducted to detect comprehension, grammatical, or semantic errors, to ensure the correct understanding of the instrument [36,37,38]. An interview protocol was designed that was replicated with all the participants. Table 1 below shows a sample of the questions utilized (see Table 1). The selection criteria for individuals to undergo cognitive interviews included being of legal age, being a family caregiver for someone with chronic conditions, and having proficiency in the Spanish language. In a random manner, the study was introduced to various family members in different outpatient areas of a medium-sized hospital in Valencia. Participants who expressed an interest in participating and met the criteria were included until the sample was saturated. For the qualitative analysis, the collection of data was conducted with the retrospective verbal probing technique [36,37,38], through pre-established questions that invited reflection on subjective aspects of the scale. The responses were recorded and transcribed afterward. The nurse explained the procedure to family caregivers in the waiting room, and those who agreed to participate signed an informed consent form; afterward, they were called for the interviews in agreed-upon private places. The results obtained were grouped according to categories and sub-categories. All the interviews were conducted, transcribed, and analyzed by the same person to ensure the homogeneity of the process. The Excel program was utilized for the categorization and analysis of the results.

Next, a quantitative pre-test was performed to explore the basic psychometric properties of the EC-PC-Family scale and to obtain preliminary information associated with it [39]. In the quantitative analysis, different basic psychometric aspects were measured, such as viability, internal consistency of the instrument, or the mean response time. The quantitative results were analyzed with the SPSS v.25 software program.

### 2.1. Data Sources

In phase 1, an ad hoc sample was constructed by selecting professionals who were experts in LTCs, as well as family caregivers of LTC patients. The family members from the pre-test phase were selected ad hoc at a medium-sized private hospital in Spain.

### 2.2. Ethical Considerations

The present study was approved by the Ethics Committee from the University of Valencia (Ref. 1648640757145). To ensure the privacy of the participant’s data, they were provided with information orally and in writing, the latter as an informational letter and an informed consent form. These forms provided an explanation of the nature of the study, their free participation, and the confidentiality of the data obtained, according to LOPD/2018, which were codified posteriorly. At the same time, the participants were informed that they could leave the study if they desired, without any problem.

## 3. Results

The creation and development of the EC-PC-Family scale went through different stages to guarantee its correct, well-defined writing, and the inclusion of valuable information about family caregivers. For example, it includes the need to not only provide care for the person with LTCs, but also themselves, or the need for the availability of social support. Next, the most important results from each phase of the process conducted are described:

1. Phase 1. Adaptation of the EC-PC scale to the family caregiver.

After the analysis of the original EC-PC, 22 of the 26 items (1, 2, 3, 4, 5, 6, 7, 10, 12, 13, 14, 15, 16, 17, 18, 19, 20, 21, 22, 24, 25, and 26) were slightly modified, changing their description and substituting phrases such as “my disease” to “the family member’s disease”, or “my state” to “the family state” [15]. Next, twelve new items were integrated to include the differences found according to the perspective of the family caregivers [7]. Version 1 (V1) of the EC-PC-Family scale was composed of 38 items. Table 2 shows an example of the changes made.

2. Phase 2: Consensus through panel of experts.

In versions V1–V3, the experts assessed the adaptation of the items, their relevance in the dimension assigned, and their understandability. After various rounds between experts and family caregivers to refine them, by including the modifications of the items that shaped the scale, a universal consensus was reached in V5 of the EC-PC-Family. Ultimately, the scale was composed of 34 items, grouped into five domains, as shown in Table 3. The most important statistical results found on the relevance, clarity, and suitability of the items that shape V5 of the EC-PC-Family scale are shown below:

- S-CVI results (relevance of the general questionnaire): The S-CVI was calculated by adding all the I-CVI divided by 34, obtaining a value of S-CVI = 0.95, while the S-CVI/UA was calculated by adding all the items equal to 1.00 (19 items), divided by 34, with the result obtained being S-CVI/UA = 0.56. These results indicate that according to the Universal Agreement method, the instrument has a moderate content validity (0.56), while the mean approach shows a high validity (0.95).
- Kappa: The Kappa values higher than 0.74 are considered excellent. All the items in V5 of the EC-PC-Family showed Kappa results >0.82 (see Table 3).
- Clarity results (individual items and general questionnaire): The mean clarity results for the individual items varied between 2.54 and 3.00. More specifically, fourteen items obtained a mean clarity score of 3.00, ten obtained a score of 2.83, eight obtained a score of 2.73, and one a score of 2.54 (see Table 3). The general clarity score of V5 of the EC-PC-Family was 2.8.
- CVR results: In this case, none of the items included in V5 of the EC-PC-Family were eliminated, with a mean CVR of 0.88 maintained. Thirteen of the items obtained a CVR of 1.00, eighteen obtained a score of 0.88, and two obtained a score of 0.64.

3. Phase 3: Pre-test of the instrument.

A qualitative pilot study through cognitive interviews was conducted with 16 family caregivers, of which 38% (6/16) were men with an average age of 60.9 years (SD ± 2.32), and the remaining 64% (10/16) were women with an average of 55.7 years (SD ± 3.6). After the verbatim transcription of the interviews, the most relevant concepts were extracted, so that 10 main themes were categorized inductively. The answers were initially grouped into 47 sub-themes, and posteriorly, after a second round of refinement, they were re-grouped into 37 sub-themes. The pilot study ended when new themes and sub-themes were no longer identified, indicating data saturation [38,40]. After the analysis of the answers, it was verified that the concepts were adequately understood, such as “living with a long term conditions”, or “become angry due to the disease of a family member”. Likewise, expressions such as “do everything possible”, or “change for good” were evaluated. This phase of the study provided results on the meaning of the concepts and expressions included in the instrument for the family caregivers, verifying their adequate meaning and understanding of the family caregivers, and therefore, the pertinent use of the EC-PC-Family. Table 4 shows an example of the work performed with each of the concepts analyzed.

In addition, the quantitative pilot study was conducted with 25 family caregivers. Of these, 64% were women (16/25), and 9% were men (9/16), with a mean age of 57.63 (±15.96); 36% (9/25) were retired. With respect to the number of hours dedicated to caregiving, 32% (8/25) did so every day for at least 8 h, and 24% (6/25) dedicated 24 h a day to the care of the family member. The preliminary results about acceptability were adequate, without the presence of ceiling and floor effects in its domains. The sample as a whole showed adequate viability, as there was no missing data. The internal consistency of the scale, as a whole, was 0.87, showing an adequate level of internal consistency, higher than that established in all the domains. The mean response time of the EC-PC-Family was 7 min, and the scale, as a whole, was assessed as clear, relevant, and useful for family caregivers.

**Figure 2:**
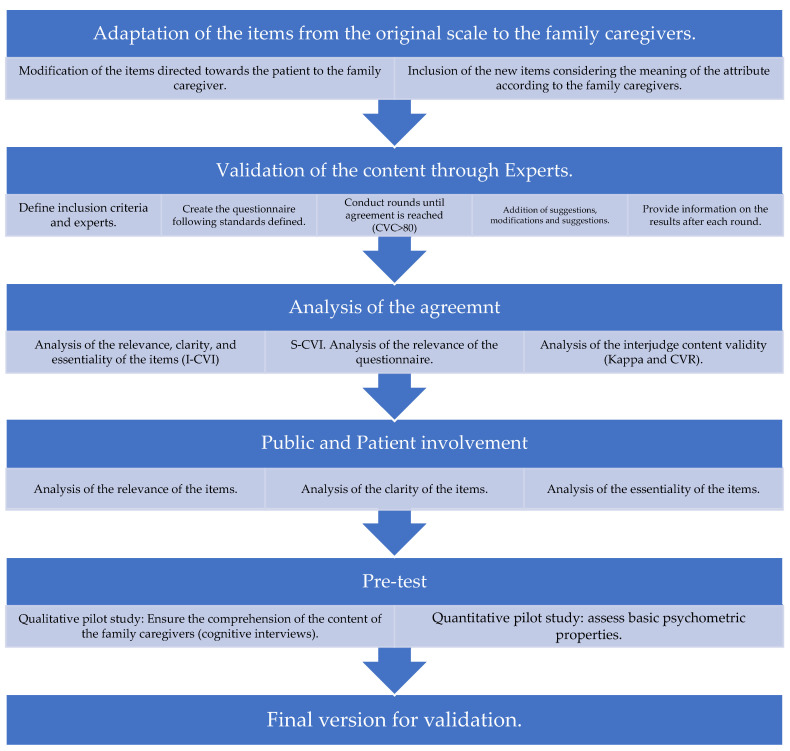
Process of adaptation of the EC-PC scale to the family caregiver. Created by the authors.

The process of adaptation of the original scale to another context was described in detail to provide further evidence of the gap found. This study illustrates the methodological steps taken from the start, starting with an already-created and validated instrument, such as the EC-PC, to adapt it to family caregivers (EC-PC-Family).This step was essential before the final validation of the scale of the study, to ensure the adequate pertinence of the items included. Each phase of the process strengthened the content and adapted and clarified the items included. Therefore, given the lack of methodological references that could have been used to guide the present process of adapting a scale that was originally directed to patients, to family caregivers, the methodological proposal used to achieve it is shown below (see Figure 2).

## 4. Discussion

The present study shows a detailed process for adapting the EC-PC scale to the family caregiver, following the good practices described by Heggestad et al. [14] for the modification of validated instruments. These well-designed scales are the basis of a great part of the understanding of a variety of phenomena, but guaranteeing that what is measured is precisely quantified, is a complex matter [41].

In our specific case, including the family caregiver’s meaning of the concept of living, which was different from the patient’s, in the dimensions of self-management and integration, was a challenge, due to the wide variety of opinions found during the consultation rounds with the experts. Nevertheless, with respect to the EC-PC-Family, an agreement was obtained in the last round, to obtain V5 of the scale (Supplementary Material available).

Transparency in the adaptation or modification of scales is a frequent source of worry in the scientific community [14,42,43], as using non-validated instruments can lead to erroneous results and conclusions [44]. In the literature, it is common to find studies in which the authors did not select adequate instruments, or utilized inappropriate instruments, considering that any modification of the original scale must be backed by a psychometric analysis of the new instrument [13,39,44,45]. In this sense, the inclusion of new items into a scale has a similar effect as the development of a new scale [14]. Therefore, during the process of adapting the EC-PC-Family scale, the changes made were detailed, as the process was similar to the construction of a new scale. The methodological gap found in the literature for the adaptation of a patient scale to a family caregiver resulted in the development of a methodological proposal that contributed towards facilitating future research studies with an approach centered on people and their close environment. Living with an LTC does not only affect the patient but the family caregivers as well, having an impact on their health and social life [3,4,5,6,12]. Therefore, in situations that are similar to living with LTCs, which affect patients and their environment, both perspectives must be included when the same phenomenon is studied. Only then will we be able to obtain results centered on the person and his or her environment, allowing us to propose and assess future interventions.

Some difficulties were found in the methodology proposed. First, contact the experts. Participating in a study with various consultation rounds is not always simple, as shown by the rejection or absence of responses from four participants who were selected at the beginning of the study. Also, with respect to the experts who agreed to participate, some difficulties were found in their response time. These results were similar to those found by other authors when applying the Delphi method [19,23,27]. In this case, the lateness in the response made it necessary to send various individualized reminders to the experts to promote their participation in each of the rounds, without a sample lost in this process. On the other hand, in the case of the family caregivers selected to participate in the process, all of them showed their commitment from the start, without requiring any reminders for their answers. In this sense, it must be underlined that the inclusion of a small sample of family caregivers in the process of adapting the scale was very enriching, as this allowed for coming close to the patient’s degree of understanding of the items [28]. Likewise, the EC-PC-Family instrument benefited from the multiple consultations and revisions of the content made by both the experts and the family caregivers, which led to the inclusion of the changes related to the concepts, writing, answer options, and the general structure of the questionnaire.

In the last stage, the scale was analyzed with a quantitative and a qualitative approach. In the qualitative part, an in-depth analysis was made of the understanding of the items that shape the scale and the subjective concepts assessed by the EC-PC-Family scale. Thus, some aspects that must be qualified in later versions were found. On the other hand, it was observed that the scale had adequate reliability as a whole and according to each of the domains separately, despite the existing limitation based on the small sample utilized.

Finally, the use of the EC-PC-Family in clinical practice will allow objectifying the degree of living with LTCs in the family caregiver. This will allow us to obtain information according to the perspectives of both parties affected by living with LTCs: the patient and the family caregiver. This information will provide social health professionals with the information necessary to become more knowledgeable on a complex, cyclical, and dynamic reality, such as living with an LTC [1,2,46]. Obtaining a comprehensive view of the phenomenon of living with LTCs could help social health professionals prevent and treat possible negative aspects derived from living with the disease, such as lack of acceptance, management, and even denial, from both patients and families. This will allow them to perform personalized evaluations of the needs and interventions centered on the person, and to provide better referrals to support systems that could be used to improve the well-being and health of the caregivers, as well as their quality of life, thereby increasing the sustainability and safety of their function.

When conducting the present study, some limitations were found. In the first place, a lack of a specific methodology for adapting the scale to a different population other than that intended with the original scale. This limitation was overcome, as shown by the detailed description of all the phases conducted for the modification of the original instrument (EC-PC), following the good practices described in the literature to perform each one of them. On the other hand, despite including a small sample of experts and family caregivers, the quantitative and qualitative studies conducted on the EC-PC-Family scale provided strength to the study. These allowed for obtaining a final version (EC-PC-Family.V5) of the clear instrument, composed of relevant and essential items for experts and users.

Including experts and family members during the in-depth analysis of the adaptation, comprehension, and meaning of the items can favor the pertinence, relevance, usefulness, and clarity of the content of the instrument. The content of various items in version 5 of the EC-PC-Family scale showed a high degree of inter-judge agreement, with a high mean content validity with respect to the clarity, relevance, and essentiality of the items that compose it. This indicates a high degree of agreement between experts and family caregivers of the items included in the scale.

Finally, the preliminary psychometric results are encouraging but future studies will have to continue delving into the psychometric properties of the EC-PC-Family through validation studies with a larger sample, with the final aim being the implementation of the EC-PC-Family scale in the practice of healthcare.

## 5. Conclusions

When a phenomenon affects both patients and their environment, such as living with LTCs, it may be necessary to include both perspectives in the tools utilized to analyze it. Only then will we be able to obtain results centered on the person and his or her environment, allowing us to propose and assess future interventions. This article elucidates the necessary methodology to adapt a scale originally designed for patient assessment to family caregivers.

The content of the items of version 5 of the EC-PC-Family showed a high index of interrater agreement. The results from this study favor the understanding of the adaptation of patient scales to family caregivers. Future studies must continue the subsequent methodological process of the EC-PC-Family V5 to contribute towards the development of knowledge in the area of measurement scales for family caregivers.

## Figures and Tables

**Table 1:** Example questions used in the cognitive interviews. Created by the authors.

Type of Question	Question Utilized
Paraphrase	In our own words, how would you define living with LTC?
Judgment/confidence	What were you thinking about when answering what are the reasons for…?
Specific	In this specific question, what is the sense of the words living with LTC?

**Table 2:** Example of the modification of the items from patient to family caregivers. Created by the authors.

EC-PC	EC-PC-Family
In my day-to-day, I have integrated the ___(LTC) and everything associated with it. For example, treatment, symptoms, changes experienced, etc.).	In my day-to-day, I have integrated the family member’s ___(LTC) and everything associated with it. For example, treatment, symptoms, changes experienced, etc.).
I know the disease and I know what I have to do to control it at all times.	I know the family member’s disease and I know what I have to do to control it at all times.

**Table 3:** V5. EC-PC-Family results consensus through panel of experts.

	Item	I-CVI ^1^ Relevance	Interpretation	I-CVI ^1^ Clarity	Interpretation	Kappa	Interpretation	CVR ^2^	Interpretation
Acceptance	1	1	Relevant	0.91	Clear	1.00	Excellent	1	Agreement
	2	0.91	Relevant	0.91	Clear	0.91	Excellent	0.82	Agreement
	3	1	Relevant	0.91	Clear	1.00	Excellent	0.82	Agreement
	4	0.82	Relevant	0.91	Clear	0.82	Excellent	0.64	Agreement
Coping	1	1	Relevant	0.82	Clear	1.00	Excellent	0.82	Agreement
	2	1	Relevant	1	Clear	1.00	Excellent	1.00	Agreement
	3	1	Relevant	0.91	Clear	1.00	Excellent	1.00	Agreement
	4	0.91	Relevant	0.82	Clear	0.91	Excellent	0.82	Agreement
	5	0.91	Relevant	0.91	Clear	0.91	Excellent	0.82	Agreement
	6	0.91	Relevant	1	Clear	0.91	Excellent	0.82	Agreement
	7	1	Relevant	1	Clear	1.00	Excellent	1.00	Agreement
Self-management	1	1	Relevant	1	Clear	1.00	Excellent	1.00	Agreement
	2	1	Relevant	0.91	Clear	1.00	Excellent	1.00	Agreement
	3	0.91	Relevant	0.82	Clear	0.91	Excellent	0.82	Agreement
	4	1	Relevant	0.91	Clear	1.00	Excellent	1.00	Agreement
	5	1	Relevant	0.73	Clear	1.00	Excellent	1.00	Agreement
	6	1	Relevant	1	Clear	1.00	Excellent	1.00	Agreement
	7	0.91	Relevant	1	Clear	0.91	Excellent	0.82	Agreement
	8	0.91	Relevant	1	Clear	0.91	Excellent	0.82	Agreement
Integration	1	1	Relevant	0.91	Clear	1.00	Excellent	1.00	Agreement
	2	0.91	Relevant	0.91	Clear	0.91	Excellent	0.82	Agreement
	3	1	Relevant	0.91	Clear	1.00	Excellent	0.82	Agreement
	4	0.91	Relevant	1	Clear	0.91	Excellent	0.82	Agreement
	5	0.91	Relevant	1	Clear	0.91	Excellent	0.82	Agreement
	6	0.91	Relevant	1	Clear	0.91	Excellent	0.82	Agreement
	7	1	Relevant	0.82	Clear	1.00	Excellent	0.82	Agreement
	8	0.91	Relevant	0.91	Clear	0.91	Excellent	0.82	Agreement
	9	0.82	Relevant	1	Clear	0.82	Excellent	0.64	Agreement
Adaptation	1	1	Relevant	1	Clear	1.00	Excellent	1.00	Agreement
	2	1	Relevant	0.82	Clear	1.00	Excellent	0.64	Agreement
	3	0.91	Relevant	0.91	Clear	0.91	Excellent	0.82	Agreement
	4	1	Relevant	0.91	Clear	1.00	Excellent	0.82	Agreement
	5	1	Relevant	1	Clear	1.00	Excellent	1	Agreement
	6	1	Relevant	1	Clear	1.00	Excellent	1	Agreement

^1^ I-CVI: Item Content Validity Index. ^2^ CVR: Content Validity Reason.

**Table 4:** Example of the conceptual analysis performed after the cognitive interviews.

Key Concepts Analyzed, Themes	Sub-Themes	Quote	Definitions after Thematic Analysis and Inductive Deduction
Meaning of Living	Pay attention to and care for another person. Help. Normalize despite the changes produced. Learn and manage the disease. Know what to do.	P2: “Continuously living with a person, and try to help by living with her”. P9. “Experience, along with the person, how the disease process is, learning and helping with whatever is needed”. P10. “Care for a person attentively” P11. “Try to manage it as best possible, and adapt to living day to day. Experience with the person the disease process, how it changes, and the worsening process”. P14 “Live in a healthy way with the disease”.	Pay attention to and care for another person, knowing what to do at each moment in time, despite the changes created in you.

## Data Availability

The data presented in this study are available on request from the corresponding author.

## Supplementary Materials

The following are available online at https://www.mdpi.com/article/10.3390/nursrep14010041/s1, Table S1. EC-PC-Family Scale (It is a draft measure not for use clinically).

## Public Involvement Statement

Family caregivers participated as experts in various phases, allowing us to elucidate the pertinence, relevance, usefulness, and clarity of the content of the instrument.

## Guidelines and Standards Statement

This manuscript was drafted following the good practices described by Heggestad et al. [14] for the modification of validated instruments.
