# Supplementary material for: Methodological Proposal for the Adaptation of the Living with Long-Term Conditions Scale to the Family Caregiver
Source: Nurs Rep. 2024 Feb 27;14(1):532–44. doi: 10.3390/nursrep14010041 (PMC10974583; doi:10.3390/nursrep14010041)
Supplement: Supplementary file 1 [file nursrep-14-00041-s001.zip › nursrep-2536786-supplementary.pdf]

V.5. EC-PC-Family Scale. **It is a draft measure not for use clinically.**

ITEM	DESCRIPTION
1	Me disgusto cada vez que pienso que mi familiar tiene esa enfermedad.
2	Intento ocultar la enfermedad de mi familiar.
3	Me molesta tener que cambiar mi forma de vivir y sacrificar cosas importantes de mi vida por la enfermedad de mi familiar.
4	Me siento culpable por la enfermedad de mi familiar.
5	Intento hacer frente a la enfermedad de mi familiar.
6	Tengo interés en buscar cosas que me motiven para no centrar mi vida solo en la enfermedad de mi familiar (como por ejemplo ir al cine, compartir experiencias con otros pacientes, pasear etc.).
7	Intento ver el lado positivo de la enfermedad de mi familiar.
8	Intento apoyarme en las cosas que son importantes en mi vida.
9	Tengo esperanza de que la situación mejore o bien, mi familiar esté en las mejores condiciones posibles.
10	Tengo a alguien que me escucha y comprende lo que estoy viviendo con la enfermedad de mi familiar.
11	Expreso mis sentimientos para que otras personas sepan cómo me siento y me ayuden.
12	Utilizo recursos (información, asociaciones, apoyo) para manejar y mejorar la situación de mi familiar.
13	Conozco la enfermedad de mi familiar y se en todo momento lo que tengo que hacer para manejarla.
14	Hago todo lo que está en mis manos para que el estado de salud de mi familiar no empeore.
15	Participo en la toma de decisiones sobre la enfermedad de mi familiar, junto a él y el equipo que le realiza el seguimiento, cuando es posible.
16	Tengo fuerza para realizar las tareas del día a día o dispongo de ayuda de forma que no me supone un esfuerzo adicional.
17	Tengo tiempo para ocuparme de mi salud.
18	Realizo algún tipo de actividad o ejercicio físico de forma habitual.
19	Presto atención a mis controles de salud y acudo a mis visitas médicas programadas.
20	He integrado en mi día a día la enfermedad crónica de mi familiar y todas las cosas relacionadas con ella (por ejemplo, tratamiento, síntomas, cambios que genera).
21	A pesar de la enfermedad de mi familiar, llevo una vida lo más normal posible.
22	Tengo en cuenta la enfermedad de mi familiar a la hora de hacer actividades, tareas y/o planes.
23	Estoy tan interesado y tengo la misma ilusión en las actividades de ocio como antes que mi familiar tuviese la enfermedad.

24	A pesar de la enfermedad de mi familiar, me siento satisfecho con mi vida.
25	Estoy satisfecho con el grado de comunicación que tengo con mi familiar, pese a la enfermedad.
26	Cuando surgen adversidades, mi familia responde de forma unida.
27	Me siento con confianza para establecer una adecuada comunicación con los profesionales sanitarios que cuidan a mi familiar.
28	En los encuentros con los profesionales de la salud, se interesan por mi estado físico y mental.
29	Gracias a la enfermedad de mi familiar ahora le doy importancia a las cosas que de verdad tienen valor.
30	La enfermedad de mi familiar, ha cambiado mi forma de ser para bien.
31	He encontrado el bienestar y armonía en mi vida.
32	A pesar de la enfermedad de mi familiar, me siento una persona igual de valiosa que otras.
33	A pesar de los problemas que la enfermedad de mi familiar genera, he encontrado un nuevo sentido a mi vida.
34	La enfermedad de mi familiar, me ha ayudado a descubrir aspectos sobre mí que desconocía.
